# Beating the exclusion rule against the coexistence of robust luminescence and ferromagnetism in chalcogenide monolayers

**DOI:** 10.1038/s41467-019-09531-0

**Published:** 2019-04-05

**Authors:** Hengli Duan, Peng Guo, Chao Wang, Hao Tan, Wei Hu, Wensheng Yan, Chao Ma, Liang Cai, Li Song, Wenhua Zhang, Zhihu Sun, Linjun Wang, Wenbo Zhao, Yuewei Yin, Xiaoguang Li, Shiqiang Wei

**Affiliations:** 1 National Synchrotron Radiation Laboratory, University of Science and Technology of China, 230029 Hefei, Anhui China; 2 Key Laboratory of Neutronics and Radiation Safety, Institute of Nuclear Energy Safety Technology, Chinese Academy of Sciences, 230031 Hefei, Anhui China; 3 College of Materials Science and Engineering, Hunan University, 410082 Changsha, Hunan China; 4 Center for Micro- and Nanoscale Research and Fabrication, University of Science and Technology of China, 230029 Hefei, Anhui China; 5 Hefei National Laboratary for Physical Sciences at the Microscale, Department of Physics, and CAS Key Laboratory of Strongly-coupled Quantum Matter Physics, University of Science and Technology of China, 230026 Hefei, Anhui China

## Abstract

Monolayer chalcogenide semiconductors with both luminescent and ferromagnetic properties are dreamed for simultaneous polarization and detection of the valley degree of freedom in valleytronics. However, a conventional chalcogenide monolayer lacks these coexisting properties due to their mutually exclusive origins. Herein we demonstrate that robust ferromagnetism and photoluminescence (PL) could be achieved in a (Co, Cr)-incorporated single monolayer MoS_2_, where the ferromagnetic interaction is activated by Co ions, and the nonradiative recombination channels of excitons is cut off by Cr ions. This strategy brings a 90-fold enhancement of saturation magnetization and 35-fold enhancement of PL intensity than the pristine MoS_2_ monolayer. The main reasons for the coexisting ferromagnetism and PL are the electronic interactions between the impurity bands of atop Cr adatoms and substitutional Co atoms, as well as the increased content of neutral exciton. Our findings could extend the applications of two-dimensional chalcogenides into spintronics, valleytronic and photoelectric devices.

## Abstract (web summary)

2D chalcogenide semiconductors with luminescence and ferromagnetism (FM) are demanded in spintronics, valleytronic and photoelectric devices. Here the authors show such characteristics in monolayer MoS_2_ by co-doping Co and Cr to build the FM interaction and cut-off the nonradiative recombination channels of excitons

## Introduction

Monolayer transition-metal dichalcogenides (TMDCs) with unit-cell thickness have emerged as a frontier for the exploration of physics and next-generation valleytronic devices, owing to the presence of an additional valley degree of freedom (DOF) that is strongly coupled with spin^1–6^. Effective manipulation and detection of the valley DOF are key issues for practical applications of monolayer TMDCs in valleytronics^1,7–10^. Currently, manipulation of the valley DOF is mainly performed under external fields such as optical, magnetic, and electrical fields. However, a very high magnetic field (beyond several Tesla) is usually required because of the non-magnetic nature of monolayer TMDCs^11–13^. When an external electric field is applied to manipulate the valley DOF, the spin-valley coupling makes it necessary to use an extra magnetic semiconductor in order to inject spin-polarized charge carriers into the non-magnetic TMDCs; this makes the valleytronic devices very complicated^14^. Besides, the efficiency of electric control of the valley DOF is severely limited by the interface between the magnetic semiconductor and TMDCs. On the other hand, detection of the polarization of the valley could be achieved by virtue of the chirality of the photoluminescence (PL) emitted by TMDCs^15,16^. Unfortunately, the optical detection is hampered by the low PL quantum yield of TMDCs obtained so far. In order to fabricate highly integrated valleytronics devices with the long dreamed capability of storing and processing information at the same time, simultaneous polarization and detection of the valley DOF would provide an opportunity. Toward this goal, monolayer TMDCs with both robust room-temperature ferromagnetism (FM) and PL will play a critical fundamental role and are highly desired.

Among a variety of TMDCs, monolayer MoS_2_ is a typical material and has attracted enormous attention due to their outstanding mechanical, optical, magnetic, and electronic properties that render them numerous potential applications. Tremendous efforts have been attempted and great progresses have been made in separately exploring and promoting the optical and ferromagnetic properties of monolayer MoS_2_^17–21^. For instance, Amani and co-workers enhanced the PL quantum yield of monolayers MoS_2_ 100 times by using a surface passivation strategy to eliminate sulphur vacancy-mediated nonradiative recombination^22^. Chemical doping of *p*-type impurities in MoS_2_ monolayer could also increase the PL intensity as a result of the reduced concentration of electron carriers^18^. Attempt to tune the PL intensity has also been made by Zhang et al.^19^ through doping the magnetic Mn ions in CVD-prepared MoS_2_ monolayer; unfortunately, the PL is actually quenched due to the formation of nonradiative recombination. Inferred from these results, high PL intensity could only be obtained in TMDCs deficient in magnetic ions and sulphur vacancy. However, the presence of abundant magnetic ions and sulphur vacancies are essential prerequisites for achieving FM of MoS_2_, as reported by Yan et al.^20^ and Andriotis et al.^21^. A dilemma then presents itself immediately. On the one hand, magnetic TM impurities or sulphur vacancy is indispensable to FM. But on the other hand, magnetic ions and sulphur vacancy tend to form defect-mediated nonradiative recombination and charged exciton (trion), both of which are detrimental to the luminescence. In other words, in analogy to the nonexistence of FM in a superconductor, the presence of FM and PL in the monolayer MoS_2_ is mutually exclusive: ferromagnetic ordering relies on the magnetic dopants and/or sulphur vacancy, which however quench the PL.

**Fig. 1:**
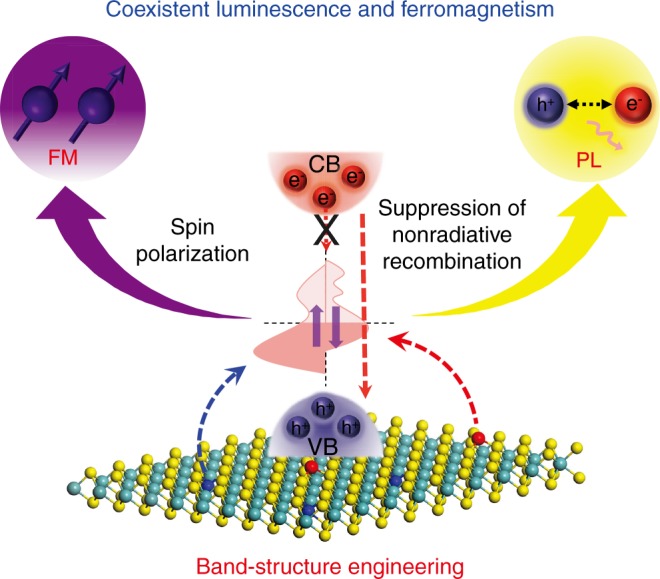
Diagrammatic representation of the coexistence principle of photoluminescence and ferromagnetism. A possible way to bring the coexistent photoluminescence and ferromagnetism to monolayer TMDCs, through a band-structure engineering that retains the spin polarization of the impurity bands while suppresses the nonradiative recombination of excitons

The dilemma above might be solved through the synergetic incorporation of two types of TMs with distinct natures. The first magnetic TM element is employed to induce the spin-polarized bandgap impurity. In many mono-doping cases, the induced impurity band with high state density is localized near the bottom of the conduction band (CB). The excited photoelectrons are apt to hop into this dense band, which decreases the probability of radiative recombination between electrons in the CB and holes in the valence band (VB): this is why the mono-doping is detrimental to the PL^23^. Provided that a second TM element capable of reducing the state density of this impurity band is incorporated, the nonradiative channel of the photo-excited electrons could be cut off and hence the radiative recombination probability could be enhanced (Fig. 1). Two delicately chosen 3*d* TM elements could be expected to meet this requirement, because their *d*–*d* electronic interactions allow for the redistribution of their impurity bands as observed in many co-doping systems^24,25^. Furthermore, as the reaction reactivity of TM elements are relevant to the electron filling of their 3*d* orbitals (below or above half-filling), TM elements of different reactivity tend to occupy different spatial positions in the chalcogenide host during the preparations of the monolayer specimen^26,27^. Therefore, there are a diversity of choices of incorporated TM elements to mediate their *d*–*d* synergetic interactions and to tailor the band structure of the monolayers, providing a flexible platform for tuning the luminescent and magnetic properties of these materials.

In this work, we propose a practical approach to bring remarkable PL and FM to TMDCs, by using two types of alien TM elements which play distinct roles in introducing magnetic moments and suppressing the nonradiative recombination of excitons. Experimentally, taking MoS_2_ monolayers as a prototype material and using the CVD method, we successfully observed both luminescent and room-temperature FM in (Co, Cr)-incorporated MoS_2_ monolayers. Detailed characterizations of the structure-property correlations unravel that the substitutional Co atoms induce a bandgap impurity band that gives rise to the ferromagnetic ordering but reduces the PL intensity because of the nonradiative recombination of excitons. After incorporating Cr atoms standing on atop sites of the monolayer, the nonradiative recombination is suppressed by the electronic interactions between the Cr- and Co-induced impurity levels. Consequently, the (Co, Cr)-MoS_2_ monolayers exhibit a 90-fold enhancement in saturation magnetization and a 35-fold increase in PL intensity relative to the pristine monolayer MoS_2_. A single flake of such a monolayer was utilized to construct a back-gate field effect transistors (FET) device. A positive magnetic resistance (MR) of 35% is observed at room-temperature. We expect that this work may extend the applications of two-dimensional chalcogenides into spintronics, valleytronic, and photoelectric devices.

## Results

### Sample preparation and characterization

The (Co, Cr)-MoS_2_ monolayer was grown on 300-nm thick SiO_2_/Si substrates prepared by chemical vapor deposition (CVD), which employed (Co, Cr)-co-doped MoO_3_ powders and pure sulphur as reactant materials (see the Methods section and Supplementary Fig. 1 for details). Compared with the case using the separate transition-metal oxides and MoO_3_ as the precursors, this method is beneficial to the homogeneous mixing of Co, Cr, and MoO_3_ at the molecular level (Supplementary Figs. 2–5)^28^. The homogeneously mixed Co and Cr atoms entering the lattice of MoO_3_ possibly adopt similar coordination structure to that of Mo atoms. Thus, the formation of (Co, Cr)-MoS_2_ nuclei avoids the precipitation of metallic Co and Cr from the preformed MoS_2_ monolayers driven by the self-purification effect. Depending on their different reactivity, Co and Cr can occupy their favorable spatial positions in the monolayer MoS_2_. Pristine monolayer MoS_2_, Co–MoS_2_, and Cr–MoS_2_ monolayers were also prepared using the CVD method.

**Fig. 2:**
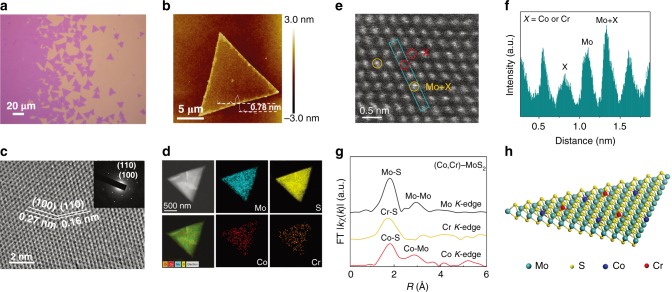
Characterization of the morphology and the occupation sites of the incorporated ions. **a** Optical image of as-prepared (Co, Cr)-MoS_2_ monolayer on 300 nm SiO_2_/Si substrates prepared by CVD. **b** AFM image, **c** HRTEM image, **d** EDX elemental mapping images, **e** HAADF-STEM image, and **f** the intensity spectra of the selected area of (Co, Cr)-MoS_2_ monolayer. **g** The FT curves of the Co, Cr *K*-edge EXAFS *k*χ(*k*) functions for (Co, Cr)-MoS_2_ monolayer_._ The Mo *K*-edge FT curve for MoS_2_ bulk reference are also displayed. **h** The atomic model of the (Co, Cr)-MoS_2_ monolayer

The morphology and crystal structure of these as-obtained monolayers are shown by the optical microscope, atomic force microscope (AFM), transmission electron microscopy (TEM) images in Fig. 2. In the optical microscope (Fig. 2a), the domains with an average size of ∼20 μm are clearly seen with a homogeneous color together with flakes having an equilateral triangular shape. The AFM image in Fig. 2b further indicates the ∼0.78 nm thickness of the as-grown flake, revealing the monolayer morphology of the as-obtained (Co, Cr)-MoS_2_ as reported in the previous studies^29,30^. As shown by the high-resolution transmission electron microscopy (HRTEM) image (Fig. 2c), the (Co, Cr)-MoS_2_ monolayer is of the hexagonal lattice structure with the lattice spacing of 0.27 and 0.16 nm assigned to the (100) and (110) planes of the 2H-MoS_2_ phase. This is further supported by the corresponding selected area electron diffraction (SAED) pattern (inset of Fig. 2c). Meanwhile, the energy-dispersive X-ray (EDX) mapping images in Fig. 2d qualitatively reveal that the chemical composition of the samples include not only the Mo and S elements, but also the Cr and Co elements. The EDX spectroscopy also indicates that besides Co and Cr, there is no other metal impurity that could induce FM (Supplementary Fig. 6). Moreover, the Mo/Co/Cr molar ratio, as determined by the inductively coupled plasma atomic emission spectrometry (ICP-AES) analysis, is ~1: 0.01: 0.003 (Supplementary Fig. 7 and Supplementary Table 1). To identify the location of Cr and Co atoms within the monolayer MoS_2_, high-angle annular dark-field scanning transmission electron microscopy (HAADF-STEM) measurements were performed. From Fig. 2e, we can see randomly distributed darker spots marked by red circles and brighter spots marked by yellow circles in the medium white (Mo atoms) atomic lattice. The corresponding cross-sectional intensity of the atom contrast in Fig. 2f reveals that the Cr or Co atoms possibly anchor on Mo atop sites or substitute for Mo atoms.

Next, X-ray absorption fine structure (XAFS) measurements were carried out to detect the real occupation positions of the Co and Cr atoms. XAFS has been a widely used technique for determination of the spatial occupations of doping elements due to its sensitivity to local atomic and electronic structures^31–33^. Figure 2g displays the Fourier transformed (FT) curve of the Cr and Co *K*-edge extended X-ray absorption fine structure (EXAFS) *k*χ(*k*) functions for (Co, Cr)-MoS_2_ monolayer. As reference, the Mo *K*-edge function of bulk MoS_2_ reference is also plotted. Evidently, the Co and Cr *K*-edge FT curves of the (Co, Cr)-MoS_2_ monolayer display considerably different features. Like the Mo *K*-edge FT curve of bulk MoS_2_, the FT at Co *K*-edge exhibits two prominent coordination peaks at 1.9 Å (Co-S coordinations) and 2.8 Å (Co–Mo coordinations), suggesting the substitutional doping of Co in the MoS_2_ host. However, only the 1.9 Å peak is prominent in the FT curve at Cr *K*-edge. In combination with the HAADF result, it can be inferred that the Cr atoms are anchored on the Mo atop sites of the monolayer MoS_2_. Based on the structural model of Co substituting for the interior Mo sites and Cr anchored on the atop Mo sites, we calculated the X-ray absorption near-edge structure (XANES) spectra at both Co and Cr *K*-edges by using the ab initio code FEFF8 (Supplementary Fig. 8)^34^. The calculations could well reproduce the spectral features of the experimental data, affording further support to the validity of the structure illustrated in Fig. 2h. These results lead us to conclude that monolayer MoS_2_ with different occupation sites of Co and Cr atoms is successfully obtained by CVD: the Cr atoms are anchored on the Mo atop sites of the monolayer MoS_2_, while Co atoms substitute for Mo in the monolayer MoS_2_ host, as indicated by the atomic model schematically shown in Fig. 2h.

### Coexistence of luminescent and magnetic properties

**Fig. 3:**
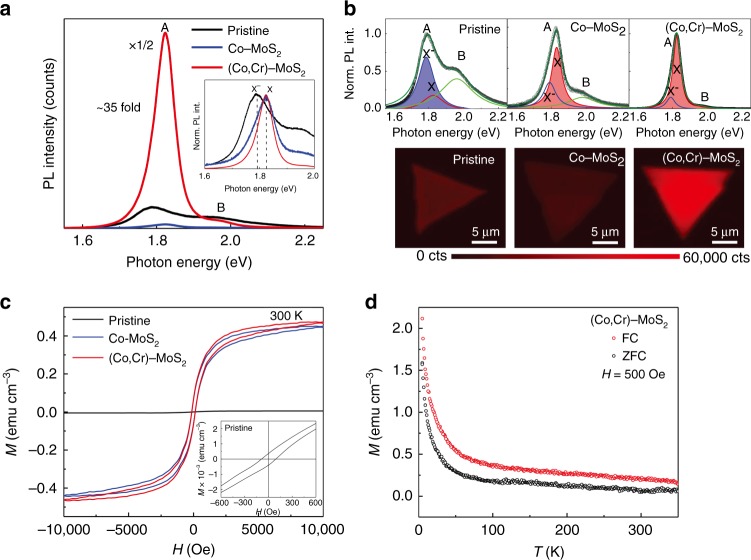
Characterization of luminescent and magnetic properties. **a** Unnormalized PL spectra of pristine monolayer MoS_2_, Co–MoS_2_, and (Co, Cr)-MoS_2_. The inset shows the normalized PL spectra. **b** Analysis of the PL spectral shapes and PL intensity mappings, and **c** Magnetization vs magnetic field (*M-H*) curves. **d** Temperature-dependence of magnetization (*M*–*T*) curves of (Co,Cr)-MoS_2_ with FC and ZFC process

To investigate the influence of Co, Cr atoms on the luminescent and magnetic properties of monolayer MoS_2_, PL spectra and magnetization (*M–H*) curves were measured at room temperature. Representative PL spectra and the integrated intensity maps of the normalized PL spectra for pristine MoS_2_, Co–MoS_2_ and (Co, Cr)-MoS_2_ monolayers are displayed in Fig. 3a, b, respectively. We first show the unnormalized PL spectra which allow us to compare directly their PL intensities. Obviously, the pristine monolayer MoS_2_ shows weak PL intensity (Fig. 3a), possibly arising from sulfur vacancies induced electrons that transform most of the excitons (X) into negative trions (X^−^) (Fig. 3b)^17^. Interestingly, the presence of sulfur vacancy, which decreases the PL intensity, induces ferromagnetic ordering (saturation magnetization of about 0.004 emu cm^−3^) in the non-magnetic pristine monolayer MoS_2_ (Fig. 3c)^20,35,36^. After Co incorporation, the saturation magnetization is greatly enhanced to ~0.4 emu cm^−3^ (0.3 µ_B_ per Co atom), 90 times higher than that of the pristine monolayer MoS_2_. And seen from Supplementary Fig. 9, the saturation magnetization of Co–MoS_2_ monolayer is decreased from ~1.0 emu cm^−3^ at 5 K to ~0.4 emu cm^−3^ at room-temperature, a feature like those observed in a diversity of transition-metal doped diluted magnetic oxides^37^. However, the PL intensity of the Co–MoS_2_ becomes weaker (Fig. 3a), in spite of the dominant exciton peak (X) (Fig. 3b). The main reason is that the substitutional Co atoms usually act as nonradiative recombination centers and significantly quench the luminescence. A similar change in the PL intensity was also observed by Zhang et al.^19^ on Mn-doped monolayer MoS_2_. Surprisingly, after incorporating Cr into the Co–MoS_2_ monolayer, the PL intensity map shows a bright red color, and the PL intensity shows a 35-fold enhancement  in relation to that of the pristine MoS_2_. Moreover, the Cr incorporation does not cause a noticeable change in the saturation magnetization (1.0 emu cm^−3^ (5 K), 0.4 emu cm^−3^ (300 K), Fig. 3c and Supplementary Fig. 9). Therefore, Cr atoms do not change the magnetic ordering of the Co–MoS_2_ monolayer, because of the low Cr content and its small contribution to the total magnetic moment. This is in agreement with the first-principles density-functional calculations by Yun et al.^38^, who predicted that doping Cr into monolayer MoS_2_ could not activate strong magnetism. From the comparison of the magnetism and PL intensity changes (Supplementary Fig. 10), we can conclude that synergetic incorporation of two TM elements can indeed realize the coexistence of outstanding optical and robust FM in monolayer MoS_2_.

**Fig. 4:**
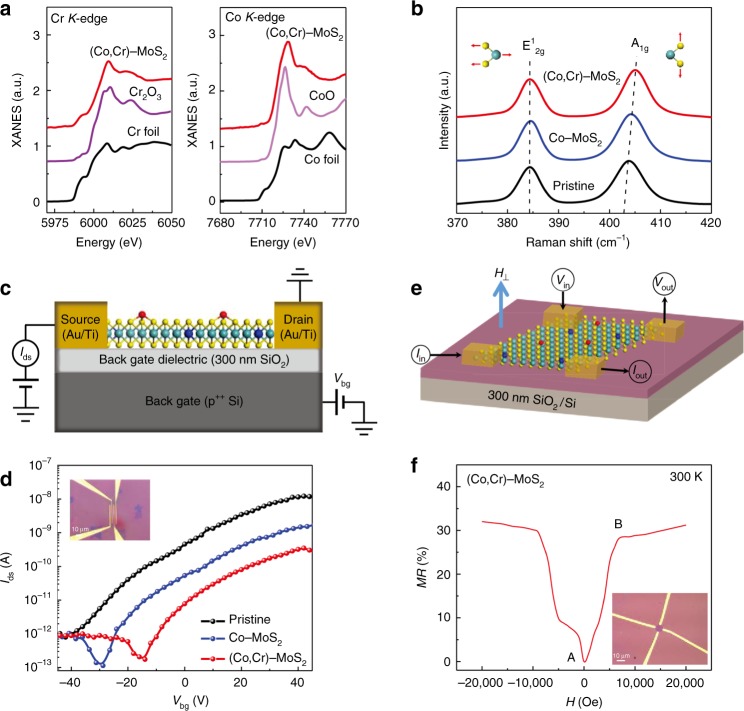
The effect of incorporating alien TM atoms into MoS_2_ monolayer. **a** Co and Cr *K*-edge XANES spectra of (Co, Cr)-MoS_2_ monolayer and Cr foil, Cr_2_O_3_, Co foil, CoO. **b** Raman spectra of pristine monolayer MoS_2_, Co–MoS_2_, and (Co, Cr)-MoS_2_. **c** Schematic illustration of the back-gate FET device. **d** Drain–source current (*I*_ds_) versus gate voltage (*V*_bg_) transfer characteristics at *V*_ds_ = 1 V. **e** Schematic illustration of van der Pawl geometry in MR measurement. **f** Magnetic-field-dependent resistance of (Co, Cr)-MoS_2_ at 300 K

To enrich our understanding of the magnetic behaviors of the doped MoS_2_, we have measured the temperature-dependence of magnetization (*M*–*T*) curves of all samples. Zero-field-cooled (ZFC) and field cooled (FC) measurements were performed on the representative samples (Fig. 3d). From the *M*–*T* and *M*–*H* data, besides the FM, diamagnetism and paramagnetism could be observed (see Supplementary Fig. 11 for details of the analysis of these magnetic components). The ZFC-FC curves display similar features to those of dilute ferromagnetic oxides and suggest their similar intrinsic magnetic behaviors^37^. More importantly, the ZFC-FC curves show the nonexistence of superparamagnetic phase transition and progressive freezing of spins in our doped MoS_2_ samples, thus excluding the precipitation of the Co clusters. If we assume that the magnetism were originated from ferromagnetic metallic Co clusters, from the measured saturation magnetization (0.3 μ_B_ per atom) and that (1.7 μ_B_ per atom) of a Co ion in Co metal clusters, we could estimate that 15% of all the doped Co atoms are in the form of Co clusters. Such a high level of Co clusters could be easily detected by XANES^39,40^. However, seen from Fig. 4a, our XANES data do not show any characteristic peaks of metallic Co cluster. From the vibrating sample magnetometer (VSM) measurements, the Curie temperature (*T*_C_) of (Co, Cr)-MoS_2_ monolayer is around 500 K (Supplementary Fig. 12), which is much lower than the *T*_C_ of metallic Co clusters. Besides, in our EDX spectroscopy and ICP-AES measurements, detectable impurity elements that may trigger the magnetism are only Co and Cr (see Supplementary Fig. 6, 7).

Summarizing the above results, it can be concluded that the magnetic behavior of our doped MoS_2_ originates from the substitutional Co ions. Due to the low Co content and the existence of sulphur vacancy, the room-temperature FM could be understood within the framework of bound magnetic polaron mechanism^41^. That is, the spins of the localized defects (electrons bond by S vacancy) align those of the nearby Co ions, producing an effective magnetic field and activating the ferromagnetic interactions between Co ions within the polaron radius (Supplementary Fig. 13). It is worthy of note that the coercive field for a single MoS_2_ layer is close to that of a large number of films. This suggests that the FM observed in a large number of films really originates from single MoS_2_ monolayers, rather than from the interactions between different MoS_2_ monolayers (see Supplementary Fig. 14 for the magneto-optical Kerr effect (MOKE) signal). It reflects the fact that the FM in a large number of MX_2_ films actually comes from the FM of a single layer.

### Effect of incorporating alien TM atoms

Next, XANES spectra were employed to understand why incorporation of Co and Cr could effectively tune the magnetic and optical properties of monolayer MoS_2_. As shown in Fig. 4a, the Cr *K*-edge XANES spectrum shows an absorption edge position quite close to Cr^3+^ in Cr_2_O_3_ but different from Cr^0^ in Cr foil, indicating the 3+ valence of Cr in the monolayer MoS_2_. On the other hand, the Co absorption edge position is close to that of CoO compound (Fig. 4a), implying the Co^2+^ valence of the substitutional Co. The Raman spectra in Fig. 4b further show that associated with the doping of Co^2+^ and Cr^3+^ ions, the A_1g_ peak (out-of-plane vibration mode) is blue shifted gradually, while the frequency of in-plane vibration mode (E^1^_2g_ peak) is almost unaffected. This suggests that the electron carrier density in monolayer MoS_2_ is reduced upon incorporation of Co and Cr^42,43^. For getting further knowledge on this point, we characterized the electrical transport in monolayer MoS_2_ before and after Co incorporation and (Co, Cr)-incorporation. The schematic illustration and optical image of the back-gate FET device by using a single flake of the (Co, Cr)- MoS_2_ monolayer with the Ti/Au (10 nm/100 nm) source and drain electrodes are shown in Fig. 4c and inset in Fig. 4d, respectively. Obviously, the threshold voltages (*V*_bg, th_) of Co–MoS_2_ (−30 V) and (Co, Cr)-MoS_2_ (−15 V) substantially shift compared to that of pristine monolayer MoS_2_ (−40 V) with lower current level, as shown in the source-drain current (*I*_ds_) versus gate voltage (*V*_bg_) transfer curves (Fig. 4d and Supplementary Fig. 15). According to the parallel-plate capacitor model, the charge concentration can be calculated from the equation *n* = *C*_ox_Δ*V*_bg_/*e*, where *C*_ox_ = ɛ_0_ɛ_r_/*d*_ox_, *e* *=* 1.602 × 10^−19^ *C* is the elementary charge and Δ*V*_bg_ = *V*_bg_−*V*_bg, th_^44^. At *V*_bg_ = 0 V, the charge concentration of the pristine MoS_2_, Co–MoS_2_, and (Co, Cr)-MoS_2_ is ~2.9 × 10^12^, 2.1 × 10^12^, and 1.1 × 10^12^ cm^−2^, respectively. Therefore, the Co and Cr in monolayer MoS_2_ act as the intrinsic *p*-type dopants and reduce the electron density. A consequence of the reduced electron density is the transition of the main part of the negatively charged trion (X^−^) into neutral exciton (X), as illustrated in Fig. 3b. Therefore, the relative increase in exciton content is the main reason for the enhanced PL intensity in the (Co, Cr)-MoS_2_ monolayer.

Based on the van der Pawl geometry, as schematically shown in Fig. 4e, under low magnetic field, the (Co, Cr)-MoS_2_ monolayer exhibits a positive MR with a surprising magnitude of 35% (Fig. 4f) at room-temperature. Besides, in the MR curve the positions of the two characteristic points A and B, which reflect the coercive field and saturation field, match well with the coercive field (~100 Oe) and saturation field (~10 kOe) determined in the out-of-plane *M*−*H* loop, respectively (Supplementary Fig. 16). It is worthy of note that the magnetic field needed for saturating the in-plane MR is lower than that for the out-of-plane MR (Supplementary Fig. 17). This is in line with the *M-H* results shown in Supplementary Fig. 16, where the saturation fields decreases as the external magnetic field is changed from the out-of-plane to the in-plane direction. These results show the magnetic anisotropy of (Co, Cr)-MoS_2_ with the easy magnetization axis parallel to the surface of monolayer. To the best of our knowledge, positive MR on MoS_2_ monolayer-based materials has been never observed before. Positive MR was previously observed on Co-doped ZnO nanowires with room-temperature FM by Yang et al.^45^, who interpreted it as a result of the electron redistribution between two FM-induced sub-bands in the presence of a magnetic field. In a single (Co, Cr)-MoS_2_ monolayer, anomalous Hall effect is observed (Supplementary Fig. 18). This measurement affords further evidence to the ferromagnetic behavior of the (Co, Cr)-MoS_2_ monolayer, and also to the magnetic interactions between the doped ions. We hypothesize that the MR is due to the broken time-reversal symmetry of the MoS_2_ monolayer by the magnetic exchange interaction between magnetic atoms, thus the two-fold valley degeneracy is lifted^13,46^. That is, the FM acts as a factor to manipulate the valley DOF as we expected. In (Co, Cr)-MoS_2_ monolayer, there emerges a large separation in the momentum space between two valleys with lifted degeneracy. At room-temperature, the energy difference between these valleys is still higher than the Fermi energy, and the difference of the electron populations in these two valleys are still remarkable. Therefore, the positive MR remains at room-temperature. In other words, the positive MR for the (Co, Cr)-MoS_2_ monolayer is intimately related to the valley polarization induced by FM property.

## Discussion

**Fig. 5:**
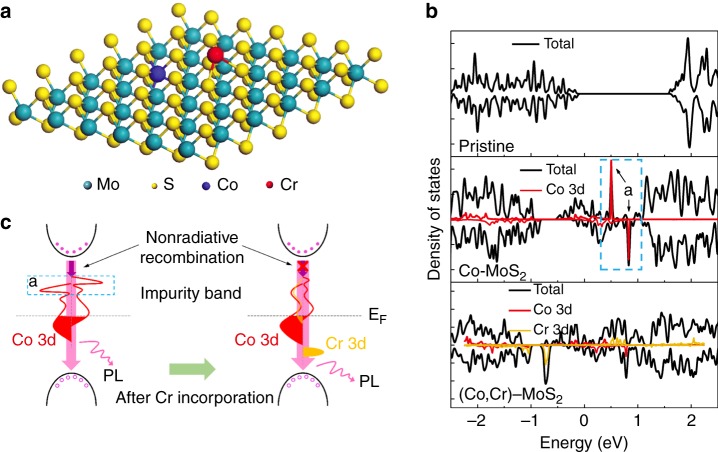
The origin of the coexisting robust magnetism and PL. **a** Structure model for the DFT calculations on the (Co, Cr)-MoS_2_ monolayer. **b** Calculated density of states (DOS) of monolayer MoS_2_: pristine MoS_2_, Co–MoS_2_, and (Co, Cr)-MoS_2_ monolayers. **c** Schematic band structure of monolayer (Co, Cr)-MoS_2_ with impurity bands

For an in-depth understanding of the origin of the coexisting robust magnetism and PL from the aspect of electronic band structure, we employed ABINIT software package to calculate the electronic structure of pristine MoS_2_, Co–MoS_2_, and (Co, Cr)-MoS_2_ monolayers (Fig. 5a). This software package implements density-functional theory using a plane-wave basis set and generalized gradient approximation (GGA) with the Perdew–Burke–Enzerhoff (PBE) functional. The details of the calculations are included in the Methods section. The obtained density of states (DOS) are plotted in Fig. 5b. The calculated bandgap of pristine monolayer MoS_2_ is 1.65 eV, underestimated by merely 0.15 eV compared with the experimental and calculated values previously^47,48^. The small difference indicates the validity of GGA for MoS_2_.

For Co–MoS_2_ monolayer, a striking bandgap impurity band can be observed, and the spin polarization contributes to magnetism with a magnetic moment of 3 µ_B_ per cell, in agreement with the result by Wang et al.^49^. The forementioned discussions indicate that the room-temperature ferromagnetic ordering of the sample originates from the substitutional Co atoms with non-zero magnetic moments. However, the substitutional Co atoms also induce a localized impurity band *a* within the bandgap (Fig. 5b). Due to the high state density, this impurity band provides a nonradiative de-exciting channel for the excited CB photoelectrons^23^. That is to say, the bandgap impurity band *a* acts as the nonradiative recombination center and reduces the PL intensity (Fig. 5c). In contrast, the impurity band induced by Cr atoms does not lead to remarkable nonradiative recombination as revealed by the PL experiment, since the PL intensity of monolayer MoS_2_ is not reduced significantly by Cr incorporation (Supplementary Fig. 19). In fact, similar phenomenon has also been observed in Cr-doped ZnO semiconductor^50,51,52^. These results indicate that Cr atoms, different from magnetic TM ions (such as Mn and Co), do not reduce the luminescence of semiconductor hosts, possibly because of the lack of high-density bandgap impurity band. Incorporating Cr with Co causes a remarkable redistribution of Co-induced bandgap impurity; especially, the state density of localized impurity band *a* is reduced significantly. Therefore, the Cr adatoms could suppress the nonradiative recombination caused by Co mono-doping (Fig. 5b, c), through electronic interactions between the Cr and Co in the MoS_2_ monolayer. Similar changes is also observed in the DOSs generated by using the local-density approximation (LDA) plus spin-orbit coupling (SOC) (Supplementary Fig. 20), in agreement with the GGA calculations. The Bader charge analysis further shows that on the average about 0.30 *e* electron is transferred from a Cr atom to the Co–MoS_2_ monolayer. On the other side, the solo-doping of Cr could not improve remarkably the PL intensity of the MoS_2_ monolayer, as inferred from Supplementary Fig. 20. Thus the synergetic incorporation of Co and Cr breaks the physical mechanism that excludes the coexistence of magnetic ordering and luminescent feature in MoS_2_ monolayer.

In conclusion, using the monolayer MoS_2_ as an example, we have experimentally demonstrated that the synergetic interaction of two alien TM elements provides an effective way to realize the coexistent optical and ferromagnetic properties in two-dimensional chalcogenide semiconductors. As shown by a detailed study of structural, optical and magnetic properties, this idea is successfully applied to MoS_2_ monolayer incorporated by Co and Cr atoms. As compared with the pristine monolayer MoS_2_, the saturation magnetization and PL intensity of the (Co, Cr)-MoS_2_ monolayer is enhanced by 90 and 35 times, respectively. A single flake of the (Co, Cr)-MoS_2_ monolayer was used to construct a back-gate FET device; the electricity measurement shows the intrinsic p-type doping of Co and Cr in MoS_2_. Based on the van der Pawl geometry, a positive MR (35%) is observed at room-temperature, which arises from the p-type doping and is intimately related to the valley polarization induced by the FM. We expect that this idea can be generalized to tune the optical and magnetic properties of other two-dimensional semiconducting materials, and it opens up possibilities for simultaneous polarization and detection of the valley DOF for future valleytronics applications.

## Methods

### Precursors prepared

To synthesize monolayer (Co, Cr)-MoS_2_, the precursors of Co–MoO_3_ and Cr–MoO_3_ were prepared by the sol-gel method. We dissolved 0.856 g of hexaammonium heptamolybdate tetrahydrate (NH_4_)_6_Mo_7_O_24_·4H_2_O, 45 mg of cobaltous nitrate hexahydrate Co(NO)_3_·6H_2_O and 10 mg of Cr(NO)_3_·9H_2_O, 2.1 g of citric acid in deionized water. The mixed solution was stirred to form sol and dried at 110 C for 2 h. Then the obtained gel was heated at 150 °C for 12 h, calcined at 550 °C for 5 h, and then cooled to room temperature naturally. The Cr–MoO_3_ was prepared in the same way, namely, a solution of 0.856 g of hexaammonium heptamolybdate tetrahydrate (NH_4_)_6_Mo_7_O_24_·4H_2_O, 10 mg of chromic nitrate nonahydrate Cr(NO)_3_·9H_2_O, 2.1 g of citric acid in deionized water was stirred to form sol and dried at 110 °C for 2 h. Then the obtained gel was heated at 90 °C for 12 h and calcined at 600 °C for 5 h.

### CVD of monolayer MoS_2_

MoS_2_ monolayers were synthesized by CVD method on 300 nm SiO_2_/Si substrates. The substrates were cleaned in acetone and isopropanol, then soaked in H_2_SO_4_/H_2_O_2_ (3:1) for 1 h and treated by O_2_ plasma for 10 min. As shown in Supplementary Fig. 1, the as-obtained MoO_3_ precursors and sulfur were loaded in two crucibles in 2-inch two-zone tube furnace, with sulfur located upstream and the substrates placed face-down above the crucible containing MoO_3_ precursors. The tube was pumped and flushed with Ar carrier gas three times and purged for 30 min with 200 sccm Ar. Then the MoO_3_ precursor zone was heated to 850 °C in 70 min with 100 sccm Ar at atmosphere pressure. When the temperature reached 650 °C, sulfur precursor zone was heated to 180 °C and the sulfur gas was introduced. After the growth was finished for 5 min, the furnaces were cooled down naturally.

### Transfer of CVD grown MoS_2_

The polymethyl methacrylate (PMMA) liquid was spin-coated on SiO_2_/Si substrate grown with MoS_2_ monolayers at 4000 rpm for 30 s, and baked at 180 °C for 2 min. Then, 1 M KOH was used to etch the SiO_2_ layer, and the obtained PMMA-MoS_2_ film was rinsed in deionized water three times. After that, the film was transferred and dried on the target substrates. The residue PMMA was washed with acetone.

### Device fabrication and measurement

The back-gated FETs devices were fabricated on 300 nm thick SiO_2_/Si chips to estimate the carrier density. The transferred MoS_2_ samples were spin-coated with PMMA at 2500 rpm for 1 min, and baked at 180 ansferred and dried on the target substrates. The rehen patterned by electron-beam lithography with a bilayer PMMA stack and developed in methyl isobutyl ketone (MIBK)/isopropanol (IPA) (MIBK: IPA = 1:3) solution for 3 min. A subsequent electron-beam deposition of Ti/Au (10 nm/100 nm) was followed and lifted-off in acetone. The devices were annealed in a vacuum tube furnace at 2500/isopropanosccm Ar flow and 10 sccm H_2_ flow for two hours. The obtained FETs were measured at room temperature under atmosphere pressure with a Lakeshore probe station equipped with Keithley 4200-SCS semiconductor characterization system. The van der Pawl geometry and Hall bar were fabricated by a two-step lithography. The patterns were obtained by O_2_ plasma etching after the first step lithography. The MR and Anomalous Hall Effect were measured in a Physical Property Measurement System (PPMS) system (Quantum Design).

### Material characterization

The TEM measurement was carried out on a JEM-2100F field emission electron microscope at an acceleration voltage of 200 kV. The high-resolution TEM (HRTEM) and corresponding EDX mapping analyses were performed on a JEOL JEMARF200F TEM/STEM with a spherical aberration corrector. High-resolution high-angle annular dark-field (HAADF) scanning transmission electron microscopy (STEM) was performed in a JEOL ARM200F with STEM aberration (Cs) corrector operated at 80 kV. The samples were transferred in nitrohydrochloric acid and diluted. Mo, Co, and Cr concentrations were determined by inductively coupled plasma atomic emission spectrometry (ICP-AES, Jarrel Ash model 955). The AFM image was taken with Veeco DI Nanoscope MultiMode V scanning probe microscope in tapping mode. The Cr *K*-edge and Co *K*-edge XAFS spectra were measured at the 1W1B beamline of the Beijing Synchrotron Radiation Facility (BSRF), China. Due to the small amount of Co and Cr dopants, XAFS data was acquired in a fluorescence geometry using 32-element germanium detector (Canberra). And the Mo *K*-edge XAFS spectra were collected in transmission mode at the BL14W1 beamline of the Shanghai Synchrotron Radiation Facility (SSRF), China. Magnetization studies were carried out using a superconducting quantum interference device (SQUID) magnetometer. Magnetic-field- and temperature-dependent magnetizations were measured by SQUID over a temperature range of 5–400 K at 500 Oe and fields up to 4 T, which were adjusted to be perpendicular and parallel to the surface of the sample, separately. Magnetization per formula unit for the monolayer was determined from the magnetization divided by the number of formula units calculated from the substrate size times the percentage of surface covered by a single layer. The diamagnetic and paramagnetic contributions were subtracted from the raw data to yield the ferromagnetic hysteresis loop. The in-plane MOKE hysteresis loops was measured by NanoMOKE2 from Quantum Design with a 50 mW (660 nm) laser source with a spot size ~5 μm.

### DFT calculation details

The DFT calculations were performed with the spin-polarized density function theory implemented in ABINIT soft package^52,53^. Electron-ion interaction was processed with the projected augmented wave (PAW) method. The GGA with the PBE functional was used to describe electron exchange-correlation. Van der Waals interaction correction was performed with DFT-D2 method^54^. A kinetic energy cutoff of 800 eV and a 5 × 5 × 1 *k*-grid for Brillouin zone sampling were adopted after detailed convergence tests. A 6 × 6 × 1 hexagonal supercell of MoS_2_ monolayer was employed to model the Co substitution and Cr adsorption according to the supercell size convergence test results. A vacuum layer of 18 Å was placed between adjacent MoS_2_ monolayers to prevent interlayer interaction. The Mo atop site which was energetically favorable adsorption site for TM ion adsorption on MoS_2_ substrates was considered. The adsorption structures were optimized with BGFS method until the maximum force on each atom was less than 0.1 meV Å^−1^. For comparison, the DOSs were also calculated using LDA plus SOC, as shown in Supplementary Fig. 20. The same reduction on state density of localized impurity band *a* upon Cr co-doping is captured, which confirms our claim that the Cr doping causes significant redistribution of Co-induced bandgap impurity. The obtained DOS is not spin polarized because when SOC was on, the up and down spin channels cannot be distinguished.

It is worthy of note that in order to examine the convergence of our GGA calculations, the DOSs were calculated for 3 × 3 × 1, 4 × 4 × 1, 5 × 5 × 1, and 6 × 6 × 1 monolayer MoS_2_ supercell with one Mo atom substituted by one Co atom and one Cr adatom attached. The corresponding Mo: Co: Cr elemental ratios are 100:11:11, 100: 6.3: 6.3, 100: 4.0: 4.0, and 100: 2.8: 2.8 for these supercells, respectively. No significant changes on the impurity bands in the bandgap is observed when the supercell was expanded from 4 × 4 × 1 to 5 × 5 × 1, and 6 × 6 × 1, which indicates that 4 × 4 × 1 supercell is large enough to eliminate the self-interaction between dopant atoms and to present the electronic modification of dilute dopants, in agreement with the previous result by Cheng et. al^55^. Therefore, the elemental concentration used in our DOS calculations for a 6 × 6 × 1 monolayer MoS_2_ supercell could well reflect the dilute dopant concentrations (1% Co, 0.3% Cr) in our samples.

### Details of XANES calculations

The structure models for Co substituting for the interior Mo sites and Cr anchored on the atop Mo sites were built up to model structural characters (Co, Cr)-MoS_2_. The atomic structures of the model was relaxed by optimizing the total energy; when the total energy convergence of up to 10^−6^ eV was achieved we calculated the XANES spectra for the finally obtained atomic structure. To achieve convergence of calculation, a model consisting of 251 atoms was used for model structure. The total scattering potentials including a fully relaxed core-hole were obtained iteratively, by successive calculations of the potential until self-consistency was reached. Based on this scattering potential, the final states of the excited photoelectron were then calculated. The Hedin–Lundqvist model of exchange potential with a 0.3 eV shift and additional broadening of 0.2 eV was used to give a closest match between the simulated and experimental spectra.

## Supplementary information

Supplementary Information

## Data Availability

The data that support the findings of this study are available from the corresponding authors upon reasonable request.
