# Supplementary material for: Beating the exclusion rule against the coexistence of robust luminescence and ferromagnetism in chalcogenide monolayers
Source: Nat Commun. 2019 Apr 5;10:1584. doi: 10.1038/s41467-019-09531-0 (PMC6451016; doi:10.1038/s41467-019-09531-0)
Supplement: Supplementary file 1 — Supplementary Information [file 41467_2019_9531_MOESM1_ESM.pdf]

Supplementary Information

Beating the exclusion rule against the coexistence of robust luminescence and ferromagnetism in chalcogenide monolayers

Duan et al.

List of content

Supplementary Figures
Supplementary Tables
Supplementary References

Supplementary Figures

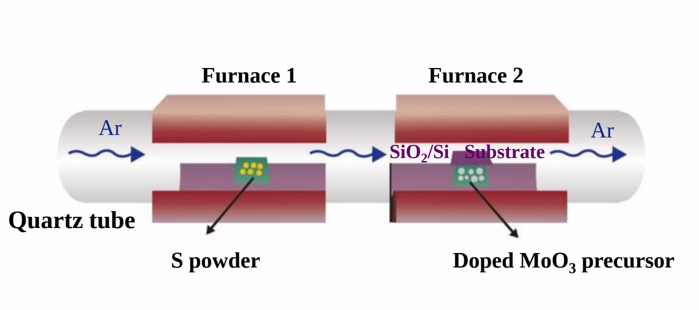

Supplementary Figure 1. Schematic illustration of the CVD process. MoS_2 monolayers were synthesized by chemical vapor deposition (CVD) method on 300 nm SiO_2/Si substrates.

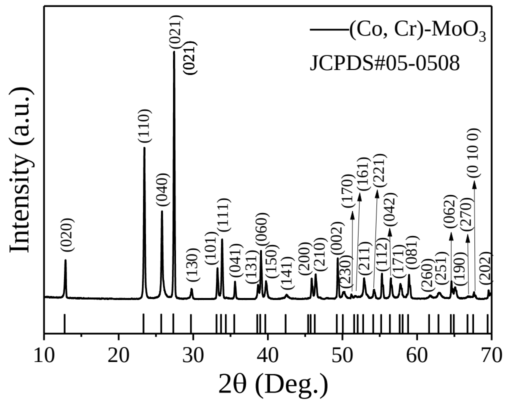

Supplementary Figure 2. XRD patterns of the (Co, Cr)-MoO_3 precursors. The X-ray diffraction (XRD) of the as-obtained MoO_3 precursors. All the diffraction peaks correspond to the α-MoO_3 with a well-crystallized orthorhombic structure.

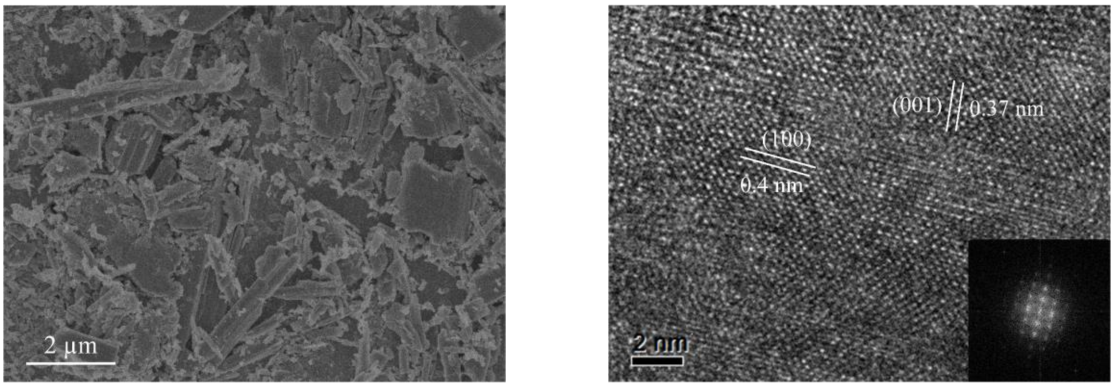

Supplementary Figure 3. SEM and TEM images of the (Co, Cr)-MoO_3 precursors. The SEM image exhibits the morphology of MoO_3 precursors. The HRTEM image further confirms the orthorhombic structure with the lattice spacing of 0.4 and 0.37 nm assigned to the (100) and (001) planes of the α-MoO_3.

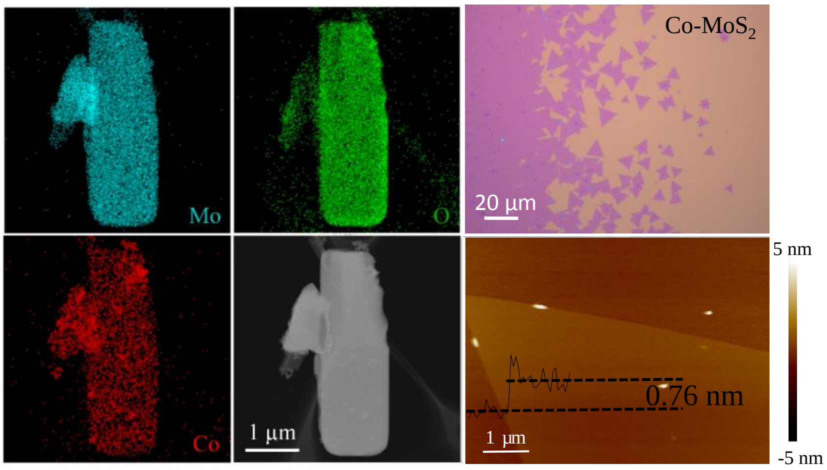

Supplementary Figure 4. EDX elemental mapping images of Co-MoO_3 precursor and the obtained MoS_2. The EDX elemental mapping images indicate the homogeneous distribution of Co atoms in Co-MoO_3.

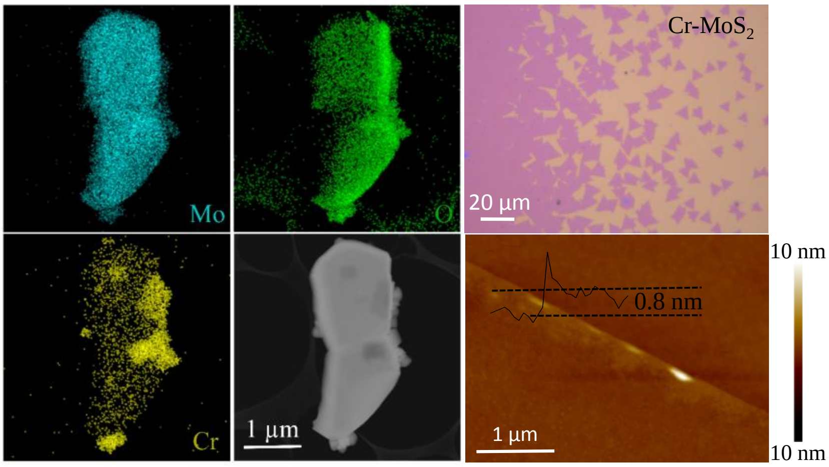

Supplementary Figure 5. EDX elemental mapping images of Cr-MoO_3 precursor and the obtained MoS_2. The EDX elemental mapping images indicate the homogeneous distribution of Cr atoms in Cr-MoO_3.

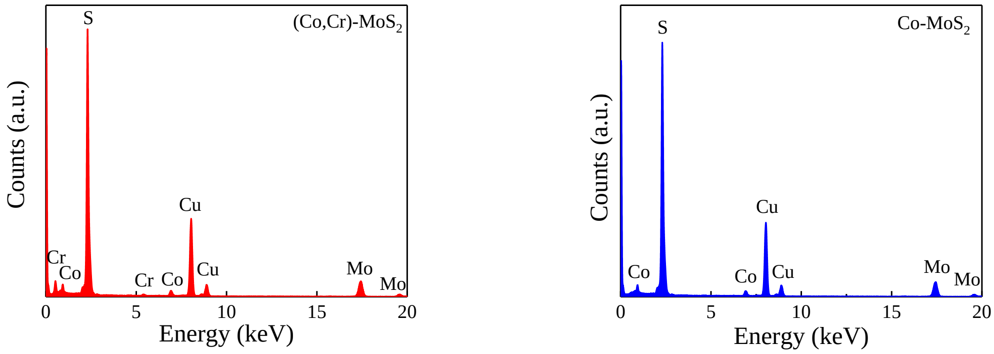

Supplementary Figure 6. Characterization of elemental composition. EDX spectra of the (Co, Cr)-MoS_2 and Co-MoS_2.

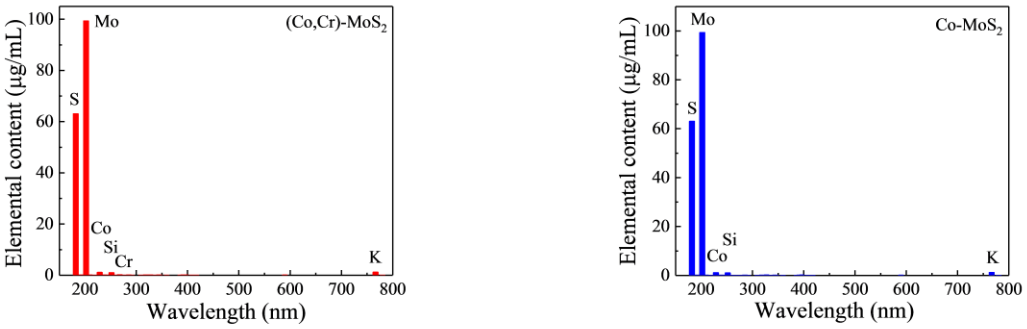

Supplementary Figure 7. Characterization of elemental composition. ICP-AES analysis of the (Co, Cr)-MoS_2 and Co-MoS_2.

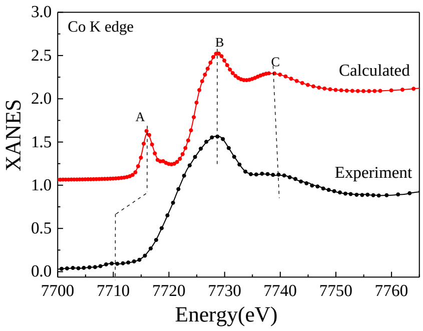

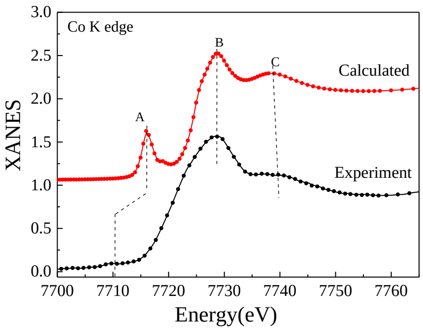

Supplementary Figure 8. **Characterization of XANES spectra.** Experimental Co and Cr *K*-edge XANES spectra of the (Co, Cr)-MoS_2, together with the calculated spectra for the structural model. The Co and Cr *K*-edge X-ray absorption near-edge structure (XANES) spectrum of (Co, Cr)-MoS_2. The Co *K*-edge spectrum exhibits three characteristic peaks A, B, and C, which could be resembled by the calculated spectrum for the structural model of Co substitution for Mo site in MoS_2 by using the ab initio code FEFF8.[2] On the other hand, the Cr *K*-edge XANES exhibits four characteristic peaks A, B, C, and D; they could be well reproduced by the calculated spectrum for Cr anchored on the atop Mo sites. These results provide support for the formation of Co substituting for the interior Mo sites and Cr anchored on the atop Mo sites.

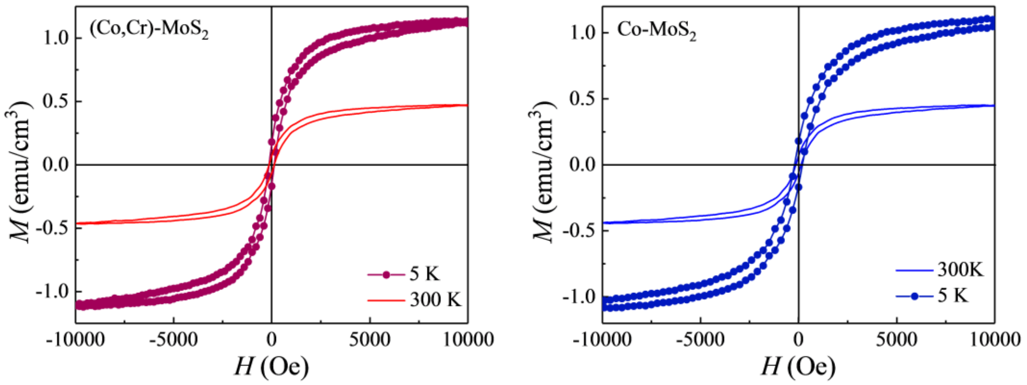

Supplementary Figure 9. Magnetization measurement. Magnetization vs. magnetic field curves at 5 K and 300 K.

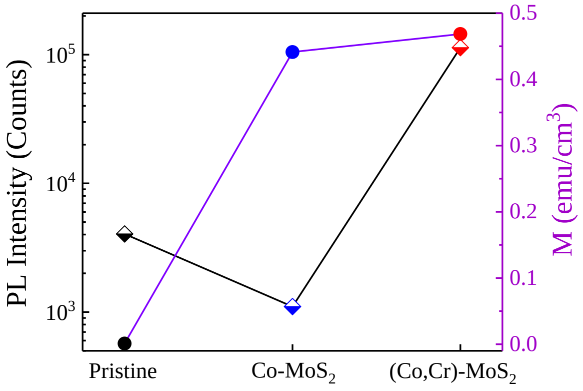

Supplementary Figure 10. Comparison of the magnetism and PL intensity. The changes of monolayer MoS_2 upon incorportaion of Co and (Co, Cr) atoms.

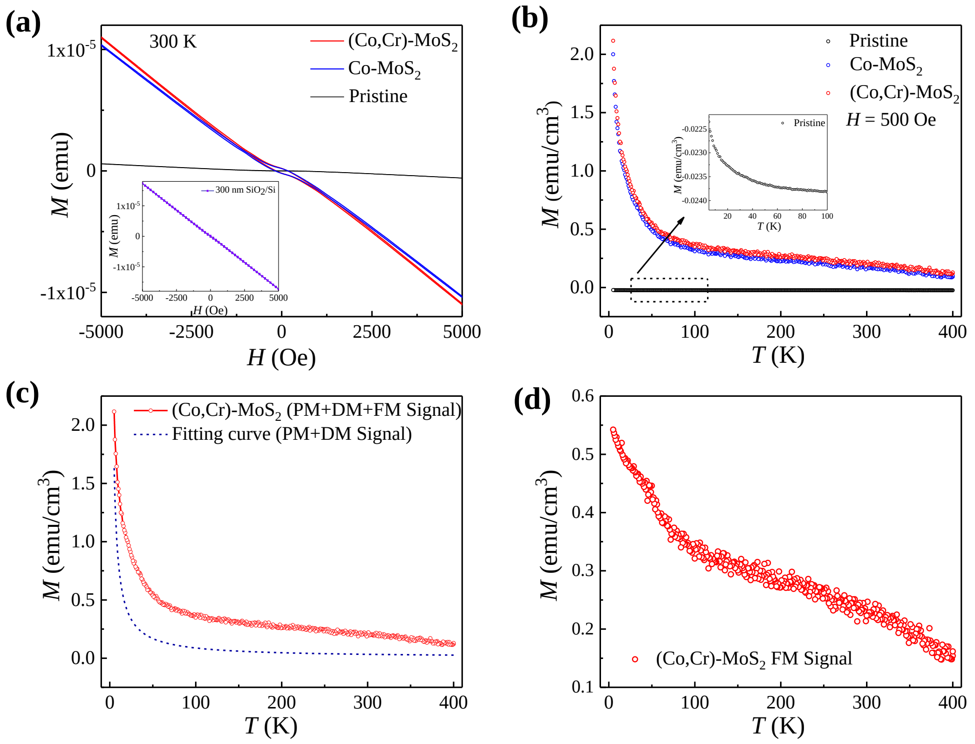

Supplementary Figure 11. Magnetization measurements. (a) Magnetization *vs* magnetic field (*M*–*H*) curves without subtracting the diamagnetic and paramagnetic background (Inset: *M*–*H* curves of 300 nm SiO_2/Si substrate indicates a diamagnetic background). (b) Temperature dependence of magnetization at 500 K. (c) The fitting result of PM+DM signals of (Co, Cr)-MoS_2. (d) The FM signal of (Co, Cr)-MoS_2 after subtracting the PM+DM signals. Supplementary Figure 11(a) displays the magnetization *vs* magnetic field (*M*–*H*) curves of the Co-MoS_2 and (Co, Cr)-MoS_2 samples. Besides the ferromagnetic (FM) component, there are also diamagnetic (DM) and paramagnetic (PM) components in both samples. The diamagnetism comes from the Si substrate and the MoS_2 host, while the paramagnetism arises from the uncorrelated spins. These magnetic components are in line with those observed by Gao et al[3] and Hank et al[4] on magnetic MoS_2 samples. Compared with the pristine MoS_2 monolayer, Co doping could substantially enhance the magnetic susceptibility (Supplementary Figure 11(b)). Taking the (Co, Cr)-doped MoS_2 sample as an example, to decompose the magnetic components (PM + DM + FM), we firstly acquired the PM + DM magnetization by fitting the M–H curves which were measured at different temperatures in a large field region. Then we fitted the temperature-dependent PM + DM magnetization ($M_{P+D}(T)$) by Curie's law: $M_{P+D}(T) = M_D + C/T$, where M_D is the DM background, C is a constant related to the applied field and Curie constant, and T is the temperature. The obtained $M_{P+D}(T)$ curves are shown in Supplementary Figure 11(c), where M_D =–0.05313 emu/cm^3; C=0.016269 emu·T/cm^3. After that, the FM signals of our samples can be extracted by subtracting the PM + DM signals from the total magnetization, as shown in Supplementary Figure 11 (d).

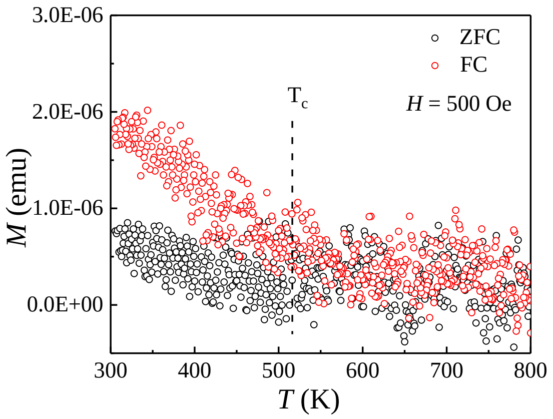

Supplementary Figure 12. **Temperature-dependence of magnetization (*M*–*T*) curves of (Co, Cr)-MoS_2 with FC and ZFC process (VSM).** Although the FC-ZFC curves obtained by VSM could not give the accurate estimation of the Curie temperature, it could still demonstrate the ferromagnetism transition at ~500 K (Supplementary Figure 12). The ferromagnetism in MoS_2 materials induced by defects, strain or alien dopants with T_C much higher than room-temperature has been previously reported in literature.[5-8] Due to the low dopant content and the low carrier concentration in our samples, the room-temperature ferromagnetism could be understood within the framework of BMP mechanism. It is widely known that the CVD preparation of MoS_2 nanosheets inevitably produces a certain amount of sulfur vacancy, which plays important roles in activating the weak ferromagnetism and PL property.[5,9,10] The spins of the localized defects (electrons bound by S vacancy) align those of the nearby Co ions, producing an effective magnetic field and activating the ferromagnetic interactions between Co ions within the polaron radius (Supplementary Figure 13).

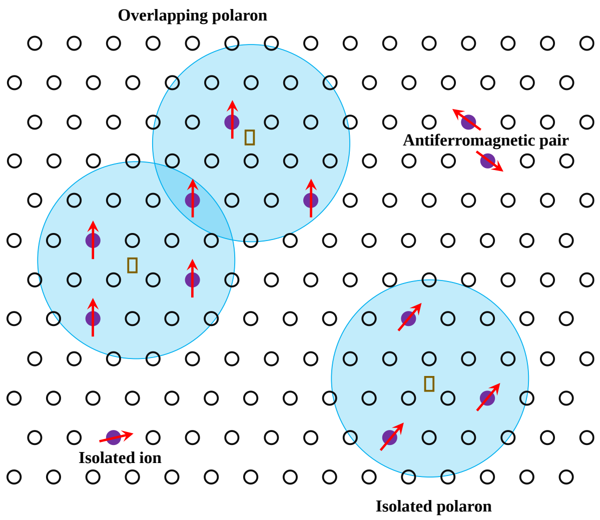

Supplementary Figure 13. Schematic presentation of magnetic polarons. Due to the low dopant content and the low carrier concentration in our samples, the room-temperature ferromagnetism could be understood within the framework of BMP mechanism. It is widely known that the CVD preparation of MoS_2 nanosheets inevitably produces a certain amount of sulfur vacancy, which plays important roles in activating the weak ferromagnetism and PL property.[5,9,10] The spins of the localized defects (electrons bound by S vacancy) align those of the nearby Co ions, producing an effective magnetic field and activating the ferromagnetic interactions between Co ions within the polaron radius.

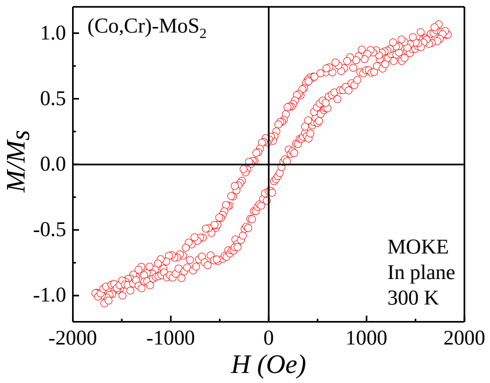

Supplementary Figure 14. Magneto-optic Kerr effect (MOKE) measurement. Our MOKE *M-H* loop measurements were performed on a single flake of (Co, Cr)-MoS_2 monolayer of about 20 μm lateral length, by using an incident light beam of several μm in size. In such a microsystem with confined space of optical elements and electromagnets, the maximum applied magnetic field is limited to only 2,000 Gauss in order to ensure the stable irradiation of the beam on the sample. Because the external magnetic field cannot saturate the (Co, Cr)-MoS_2 monolayer, the obtained M-H loop looks different from the M-H loop obtained by SQUID where the applied magnetic field could be as high as 40,000 Gauss. Such a difference has also been reported in literature. For instance, Bonilla et al. reported the seemingly different M-H loops from their SQUID and MOKE measurements on VSe_2 monolayers.[12] Nevertheless, the MOKE M-H loop still indicates unambiguously the room-temperature ferromagnetism of our single flake of (Co, Cr)-MoS_2 monolayer, and helps to rule out the possibility of the macroscopic ferromagnetism observed by SQUID arising from the interfacial interactions between different monolayers.

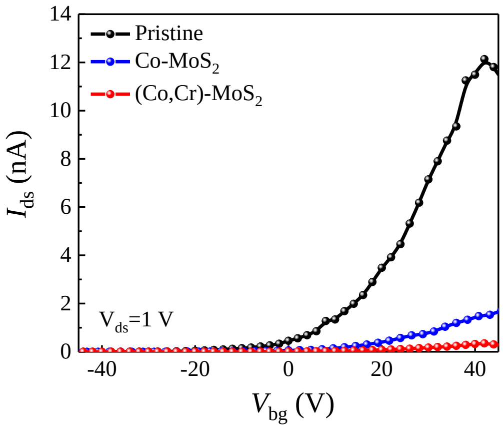

Supplementary Figure 15. **Room-temperature transfer characteristics of the back-gate FETs at V_{ds}=1V.** The electrical transfer curves showed in Supplementary Figure 16 indicate the typical n-type channel of all the FET devices. The on/off ratio of the Co-MoS_2 and the (Co, Cr)-MoS_2 decrease dramatically compared with the pristine monolayer MoS_2, mainly because of the scattering of charge impurity upon incorporating Co and Cr atoms.[11]

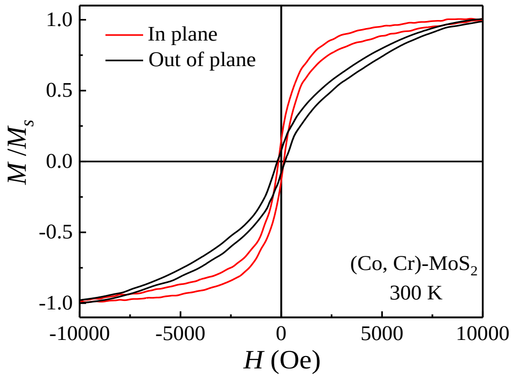

Supplementary Figure 16. Magnetization measurement. The in-plane and out-of-plane *M*-*H* loops at 300 K for (Co, Cr)-MoS_2.

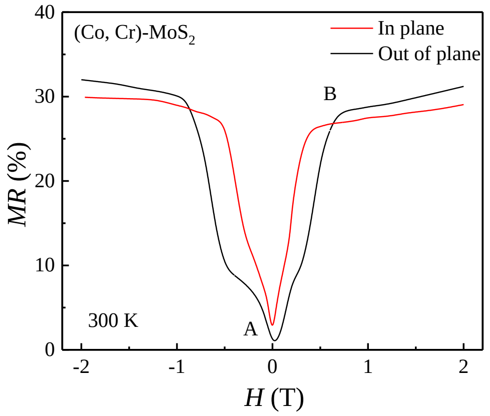

Supplementary Figure 17. **MR measurement.** Comparison of the in-plane and out-of-plane MR data of (Co, Cr)-MoS_2. Clearly, in comparison with the out-of-plane MR data, the magnetic field needed for saturating the in-plane MR is lower. This is in line with the M-H results shown in Supplementary Figure 14, where the saturation fields decreases as the external magnetic field is changed from the out-of-plane to the in-plane direction.

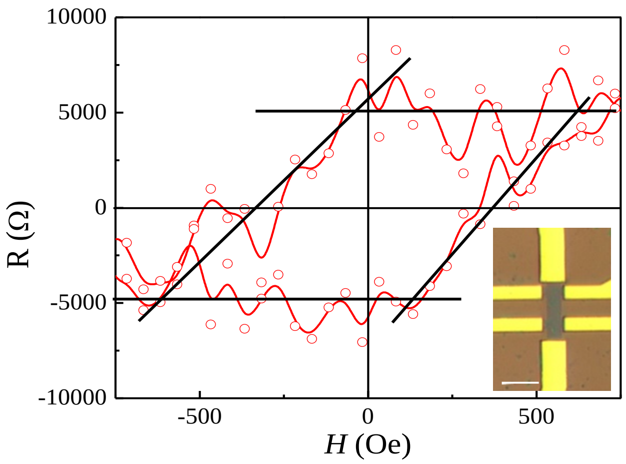

Supplementary Figure 18. Anomalous Hall-effect measurements. Magnetic field dependence of Hall resistance of (Co, Cr)-MoS_2. Scale: 5 μm. AHE is an important tool to characterize the ferromagnetic properties of materials. According to the formula $\rho_{xy}=R_0B+4\pi R_sM$, where R_0B is the ordinary Hall effect, $4\pi R_sM$ is the anomalous Hall effect with M being the magnetization, the AHE signal could be well correlated with the magnetization. It is reasonable to expect that the saturation field of AHE should be consistent with the M-H loops. In reality, however, AHE signals could be hardly detected in many ferromagnetic materials, because of the low concentrations of carriers or high magnetoresistance that make the AHE signals buried in the background signal of the AHE measurement system and even become undetectable.[13-16] For example, as reported by Huang et. al., V-doped ZnO shows the discernible M-H curves in the SQUID measurement, but no clear AHE signal was observed.[13] Oyosaki et al. also reported the absence of AHE in $Ti_{0.97}Co_{0.03}O_{2-\delta}$ films with a low carrier content.[15] In these studies, the results of AHE could not simply equate with the M-H loops. Xu et al. observed the AHE signal in Co-doped ZnO, but the signal-to-noise ratio is too poor to give a consistent saturation field with that observed in MR.[16] In our samples, since the content of the carriers is very low, the AHE signal is observable but very weak, which is not precisely equal to the M-H loops (Supplementary Figure 14) and the MR results in terms of the saturation field. In other words, we could not determine the saturation field merely from the AHE signal, and therefore the AHE results are not quantitatively equal to the M-H loops and MR results. Qualitatively, the AHE signal, weak as it is, still affords evidence to the room-temperature ferromagnetism of a single flake of (Co, Cr)-MoS_2 monolayer, in line with the MOKE M-H loops.

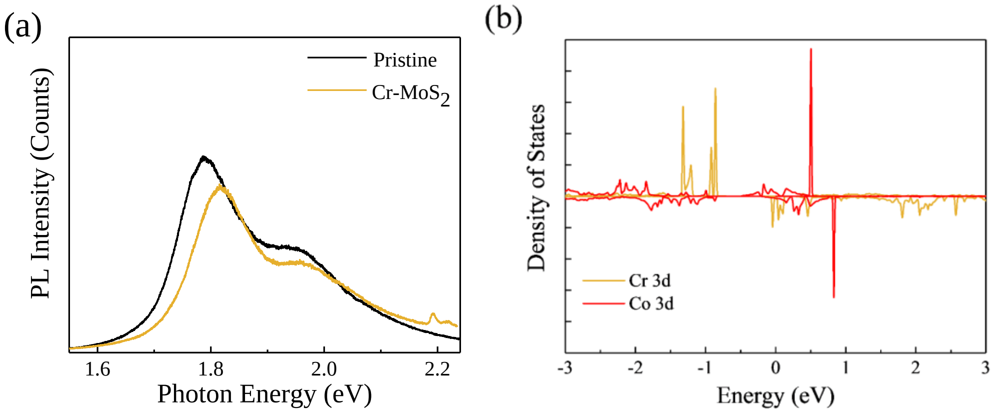

Supplementary Figure 19. **PL spectra and calculated density of states (DOS) of Cr 3d and Co 3d.** (a) PL spectra of monolayer pristine MoS_2 and Cr-MoS_2. (b) The comparison of calculated density of states (DOS) of Cr 3d and Co 3d. The PL intensity of monolayer MoS_2 is not reduced significantly by alien Cr atoms, in contrast with Co incorporation. Possibly owing to the lack of high-density bandgap impurity band compared with Co incorporation, Cr dopants does not lead to remarkable nonradiative recombination.

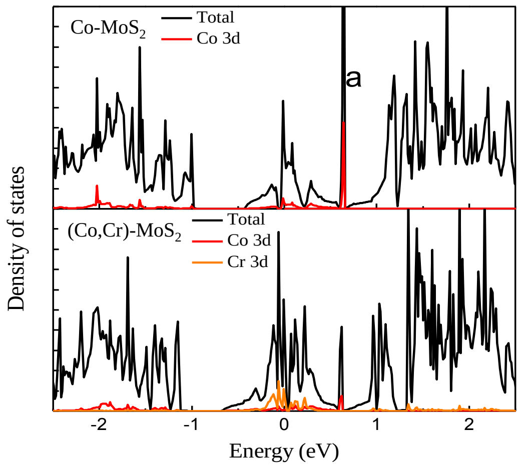

Supplementary Figure 20. **DOS calculations.** The DOS of Co-MoS_2 and (Co, Cr)-MoS_2 with LDA+SOC.

Supplementary Tables

Supplementary Table 1. Elemental Analyses of the (Co, Cr)-MoS_2 using ICP.

Name	Atomic Ratio of Mo/Co/Cr By ICP
(Co, Cr)-MoS_2	1:0.01:0.003

Supplementary References

1. Ankudinov, A. L., Ravel, B., Rehr, J. J. & Conradson, S. D. Real-space multiple-scattering calculation and interpretation of X-ray absorption near-edge structure. *Phys. Rev. B* **58,** 7565-7576 (1998).
2. Gao, D. Q. *et al.* Realization of high Curie temperature ferromagnetism in atomically thin MoS_2 and WS_2 nanosheets with uniform and flower-like morphology. *Nanoscale* **7,** 650–658(2015).
3. Han, S. W, *et al.* Controlling Ferromagnetic Easy Axis in a Layered MoS_2 Single Crystal. *Phys. Rev. Lett.* **110,** 247201 (2013).
4. Han, S. W. et al. Controlling ferromagnetic easy axis in a layered MoS_2 single crystal. *Phys. Rev. Lett.* **110,** 247201 (2013).
5. Zhang, J. et al. Magnetic molybdenum disulfide nanosheet films. *Nano Lett.* **7,** 2370-2376 (2007).
6. Yang, Z. et al. Realization of high Curie temperature ferromagnetism in atomically thin MoS_2 and WS_2 nanosheets with uniform and flower-like morphology. *Nanoscale* **7,** 650-658 (2015).
7. Xia, B., Guo, Q., Gao, D., Shi, S. & Tao, K. High temperature ferromagnetism in Cu-doped MoS_2 nanosheets. *J. Phys. D: Appl. Phys.* **49,** 165003 (2016).
8. Mak, K. F. et al. Tightly bound trions in monolayer MoS_2. *Nat. Mater.* **12,** 207 (2013).
9. Zheng, H. et al. Tuning magnetism of monolayer MoS_2 by doping vacancy and applying strain. *Appl. Phys. Lett.* **104,** 132403 (2014).
10. Radisavljevic, B. *et al.* Single-layer MoS_2 transistors. *Nat. Nanotech.* **6,** 147-150 (2011).
11. Bonilla, M. et al. Strong room-temperature ferromagnetism in VSe_2 monolayers on van der Waals substrates. *Nat. Nanotech.* **13,** 289 (2018).
12. Liu, S. et al. Reduced room-temperature ferromagnetism in intermediate conducting regime of V doped ZnO. *Appl. Phys. Lett.* **96,** 262504 (2010).
13. Shim, W. et al. Evidence for carrier-induced ferromagnetic ordering in $Zn_{1-x}Mn_xO$ thin films: Anomalous Hall effect. *J. Appl. Phys.* **101,** 123908 (2007).
14. Toyosaki, H. et al. Anomalous Hall effect governed by electron doping in a room-temperature transparent ferromagnetic semiconductor. *Nat. Mater.* **3,** 221 (2004).
15. Xu, Q. et al. Metal-insulator transition in Co-doped ZnO: Magnetotransport properties. *Phys. Rev. B* **73,** 205342 (2006).
